# Investigation of Chloride Binding Abilities of Symmetric Squaramides

**DOI:** 10.1021/acsomega.5c13503

**Published:** 2026-03-23

**Authors:** Serap Mert, Sude Minel Özger, Ayşenur Vardar, Özden Erdebil

**Affiliations:** † Department of Chemistry, Faculty of Arts and Sciences, 52980 Kocaeli University, Kocaeli 41001, Turkey; ‡ Department of Polymer Sci. and Technol., Institute of Natural and Applied Sciences, Kocaeli University, Kocaeli 41001, Turkey; § Center for Stem Cell and Gene Therapies Res. and Pract., Kocaeli University, Kocaeli 41001, Turkey; ∥ Department of Chemical Engineering, Engineering Faculty, Kocaeli University, Kocaeli 41001, Turkey

## Abstract

In this work, three symmetric squaramide derivatives were synthesized, and their anion-binding properties were systematically investigated. The binding properties of squaramides **I**–**III** with anions were determined by spectroscopic titration in DMSO-*d*_6_ using tetrabutyl ammonium salts. During titration with ammonium salts, the shift values of the protons of NH groups in the squaramide structure were recorded in ^1^H NMR. The variations in the chemical shifts of NH protons in the squaramides were analyzed with the DynaFit and BindFit programs to obtain the corresponding association constants. The greatest chemical shift was obtained from TBA-Cl with squaramide **I** titration, and it has the highest binding capacity with squaramide **I** and Cl^–^ anion. Therefore, it was found that both the number of CF_3_ groups on the aromatic ring and the number of CH_2_ groups between the aromatic ring and the amide moiety in the structure of squaramide influence the strength of the anion binding ability. The stoichiometry of complexation between squaramides **I**–**III** and Cl^–^ was also investigated using Job’s plots.

## Graphical abstract

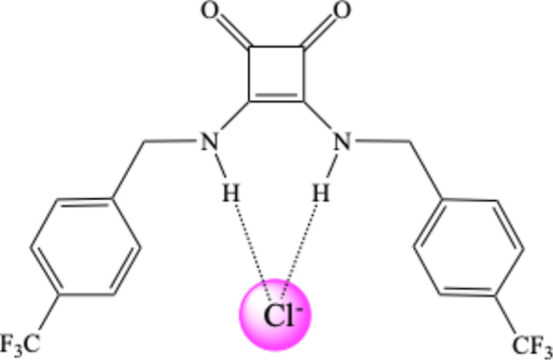

## Introduction

Anions and their interactions are widely studied in organic synthesis, disease control, environmental, and industrial applications.^1–3^ Early studies on anion binding in the literature demonstrated that the methoxide anion can be chelated with a diboron ligand,^4^ and halides can be coordinated by macrobicyclic ammonium cages^5^ through the formation of H-bonds in the cavity. The existence of a hydrogen bond donor group such as an amine and amide in the anion binding is important to interact with the anion.^6^ Squaramides^7^ are strategically placed on the structure to create highly effective anion sensors/recognition because the acidic NH groups of squaramides exhibit high affinity to anions.

Approximately 30 years ago, Tomas and co-workers reported that the addition of various “onium” salts, such as benzyl trimethylammonium (BTA) bromide or tetramethylammonium (TMA) acetate, to a chloroform solution of squaramide caused characteristic upfield shifts of the methyl and methylene protons of TMA and BTA in ^1^H NMR.^7^ They further observed that adding TMA to a dilute squaramide solution led to detectable changes in the protons of the phenol ether ring present in the squaramide structure. In a subsequent study, this research team^8^ designed a new series of tripodal squaramide-based receptors to enhance cooperative interactions with anions in the presence of ammonium salts. Later, they developed squaramide receptors containing one, two, or three squaramide subunits for carboxylate binding, and the resulting complexes were analyzed by NMR spectroscopy.^9^ In addition, computational investigations into the aromatic properties of squaramide-anion complexes demonstrated that the squaramide unit can act as both a hydrogen-bond donor and acceptor.^10^ Studies reported in the literature^7–12^ are very important to demonstrate that squaramides can be used as anion binders and have pioneered dozens of squaramide anion binding studies^13–30^ to date. Moreover, several review studies^31–34^ have summarized the use of various squaramides as receptors in this field over time.

One of the anions or a set of various anions, such as their tetrabutyl ammonium (TBA) salts of fluoride, chloride, bromide, iodide, hydrogensulfate, sulfate, hydrogenpyrophosphate, and dihydrogen phosphate, or triethylammonium hydrogen carbonate, are used to show the anion binding properties of squaramides.^7–30^ Among these anions, the binding of Cl^–^ anion, owing to its chemical, environmental, physiological, and biological importance, has been extensively studied with various squaramides.^13,16,22,23,27,30^

The aim of this study is to systematically examine how structural variations in squaramide receptors influence their Cl^–^ anion binding properties. Specifically, the effects of (i) the number of CF_3_ substituents on the aromatic ring and (ii) the length of the methylene spacer between the NH group and the aromatic ring in squaramides on Cl^–^ anion binding affinity were investigated. The incorporation of an alkyl spacer was motivated by previous studies,^34,35^ indicating that increased conformational flexibility may enhance anion-binding performance. Within this context, we report the one-step synthesis of three symmetric squaramides (Figure 1), one of which (squaramide **III**) is, to the best of our knowledge, a previously unreported derivative. Furthermore, this work presents the first detailed evaluation of Cl^–^ anion binding affinities of these squaramides in the literature. ^1^H NMR spectroscopic titrations were conducted between TBA-Cl salt (guest) as a Cl^–^ anion source and squaramides **I**–**III** (hosts) separately to evaluate their Cl^–^ anion binding abilities. Then, the association constants (*K*_a_) were determined using DynaFit and BindFit fitting tools, based on variation in the NH proton signals of squaramides in ^1^H NMR spectroscopy. In addition to TBA-Cl, ^1^H NMR spectroscopic titrations were carried out with TBA-Br, TBA-I, and TBA-perchlorate salts (guests), separately with squaramide **I** (host), which exhibited the highest *K*_a_ value for Cl^–^ anion. Moreover, *K*_a_ values for squaramide **I** and Br^–^ anion were calculated using DynaFit and BindFit programs (Supporting Information, Figures S11 and 12). However, no measurable variation on the NH proton signals was observed during titrations with TBA-I or TBA-perchlorate (data not shown); therefore, *K*_a_ values for squaramide **I** with iodide and perchlorate anions could not be obtained. Additionally, Job plot analyses were carried out to examine the complexation stoichiometry of squaramides **I**–**III** with the Cl^–^ anion and squaramide **I** with the Br^–^ anion (Figure 8).

**Figure 1:**
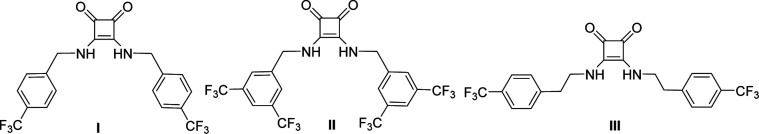
Structures of squaramides **I**, **II**, and **III**.

## Materials and Methods

### Materials

(4-(Trifluoromethyl) phenyl)­methanamine (TCI, 98%), (3,5-bis­(trifluoro methyl)­phenyl)­methanamine (Acros Organics, 95%), 2-(4-(trifluoromethyl)­phenyl) ethylamine (TCI, 98%), 3,4-dimethoxy-3-cyclobutene-1,2-dione (TCI, 98%), and dichloromethane (Sigma/Aldrich, 99.9%) were utilized for the syntheses of squaramides. Tetrabutylammonium salts such as chloride (TBA-Cl, Sigma/Aldrich), bromide, iodide, and perchlorate (TBA-Br, TBA-I, and TBA-ClO_4_, TCI Europe) were utilized as anion sources for ^1^H NMR spectroscopic titrations. [*d*_6_]­DMSO (Eurisotop) was used as a deuterated solvent for ^1^H NMR spectroscopic titrations.

### Instruments

ATR-FTIR analyses were carried out by an ATR Bruker-Tensor 27 spectrometer with a range of 4000 to 600 cm^–1^, a resolution of 4 cm^–1^, and 30 scans for each measurement. Characterization of the synthesized products and spectroscopic titrations were carried out by a Bruker Avance-III 400 MHz NMR spectrometer. Mass analyses of novel squaramide **III** were performed with an LC/MS-TOF spectrometer using an HPLC unit with 1260 Infinity series. Melting points (Mp) of the solid squaramides were measured by the Stuart brand SMP3.

### Synthesis of Squaramide **I** (3,4-Bis­(((4-trifluoromethyl)­benzyl)­amino)­cyclobut-3-ene-1,2-dione)

Commercially available 3,4-dimethoxy-3-cyclobut-3-ene-1,2-dione (71 mg, 0.5 mmol) and 4-(trifluoromethyl)­benzylamine (143 μL, 1 mmol) were stirred at rt for 48 h under an argon atmosphere. Then, squaramide **I**, as reported in the literature, was isolated by crystallization from cold methanol.^36^ Yield: 190 mg (89%). A color change was observed at approximately 247 °C, followed by darkening at around 300 °C and then melting at 326 °C. ATR-FTIR (*v*_max_/cm^–1^): 3160.33, 3062.19, 2948.66, 1802.29, 1648.13, 1566.19, 1485.67, 1428.09, 1326.51, 1246.93, 1159.12, 1114.75, 1065.64, 1019.52, 940.60, 847.71, 811.60, 761.22, 736.01, 702.86, 636.43, 594.56, 515.75. ^1^H NMR (400 MHz, DMSO-*d*_6_): δ 7.88 (br s, 2H, NH), 7.72 (s, 4H), 7.51 (s, 4H), 4.80 (s, 4H).^13^C NMR (101 MHz, DMSO-*d*_6_): δ 182.87, 167.74, 143.88, 128.14, 125.64, 122.94, 120.24, 46.27 (Figures S1–S3).

### Synthesis of Squaramide **II** (3,4-Bis­(((3,5-trifluoromethyl)­benzyl)­amino)­cyclobut-3-ene-1,2-dione)

Commercially available 3,4-dimethoxy-3-cyclobut-3-ene-1,2-dione (71 mg, 0.5 mmol) and 3,5-bis­(trifluoromethyl)­benzylamine (243 mg, 1 mmol) were stirred at rt for 48 h under argon. Then, squaramide **II**, as reported in the literature, was isolated by crystallization from cold methanol.^36–38^ Yield: 200 mg (71%). A color change was observed at around 251 °C, followed by melting at 256 °C. ATR-FTIR (*v*_max_/cm^–1^): 3156.34, 2940.49, 1804.26, 1654.24, 1564.24, 1490.28, 1445.95, 1383.10, 1334.56, 1279.72, 1126.24, 962.15, 891.33, 835.00, 761.11, 682.15, 639.13, 600.23, 563.09, 516.29, 463.50. ^1^H NMR (400 MHz, DMSO-*d*_6_): δ 8.02 (d, *J* = 5.7 Hz, 8H), 4.88 (s, 4H). ^13^C NMR (101 MHz, DMSO-*d*_6_): δ 182.98, 167.78, 142.59, 130.48 (q, *J* = 33 Hz), 128.38, 123.31 (q, *J* = 274 Hz), 121.19, 45.82 (Figures S4–S6).

### Synthesis of Squaramides **III** (3,4-Bis­((4-(trifluoromethyl)­phenethyl)­amino)­cyclobut-3-ene-1,2-dione)

Commercially available 3,4-dimethoxy-3-cyclobut-3-ene-1,2-dione (426 mg, 3 mmol) and 2-(4-trifluoromethylphenyl)­ethylamine (953 μL, 6 mmol) were stirred at rt for 48 h under argon. Then, squaramide **III**, as a novel compound, was isolated by crystallization with ethyl alcohol in 95% yield. A color change was observed at around 248 °C, followed by darkening at around 320 °C and melting at 331 °C. ATR-FTIR (*v*_max_/cm^–1^): 3149.97, 2942.65, 1800.60, 1641.60, 1561.86, 1473.48, 1430.94, 1360.44, 1320.46, 1237.43, 1201.08, 1162.96, 1119.19, 1064.24, 1012.77, 786.92, 649.83, 598.73, 513.83. ^1^H NMR (400 MHz, DMSO-*d*_6_): δ 7.65 (d, *J* = 8.0 Hz, 4H), 7.44 (d, *J* = 7.7 Hz, 4H), 7.31 (br s, 2H, NH), 3.75 (s, 4H), 2.91 (s, 4H). ^13^C NMR (101 MHz, DMSO-*d*_6_): δ 182.60, 167.78, 148.74, 143.67, 129.69, 128.53, 127.16 (q, *J* = 31 Hz), 125.82, 125.25, 123.12, 44.18, 36.83. HRMS: C_22_H_18_F_6_N_2_O_2_ ([M + H]^+^) calcd, 457.1306: found, 457.13473 (Figures S7–S10).

### NMR Titration Experiments

For the titration experiments, a 0.055 M solution of the squaramide host in [*d*_6_]­DMSO was prepared. Subsequently, aliquots of a 1.0 M solution of TBA-Cl in [*d*_6_] DMSO were added to provide 0.2–20 equiv of the anionic guest relative to the host. After each incremental addition of the anion, the ^1^H NMR spectra were recorded to monitor the chemical shift variations of the NH proton resonances for squaramides **I**–**III**. The titration procedure employed for the TBA-Cl experiments was analogously applied to TBA-Br, TBA-I, and TBA-ClO_4_ with squaramide **I**, respectively.

## Results and Discussion

### Synthesis of Squaramides

The squaramides **I** ^36^ and **II** ^36–38^ were prepared from the corresponding amines following literature procedures reported previously (Scheme 1). Moreover, novel squaramide **III** was synthesized successfully (Scheme 1). The characterizations of squaramides **I**–**III** were performed by ATR-FTIR, ^1^H, and ^13^C NMR analyses (Supporting Information, Figures S1–S9). Moreover, the successful synthesis of novel Squaramides **III** was confirmed according to mass analyses (Supporting Information, Figure S10).

**Scheme 1:**
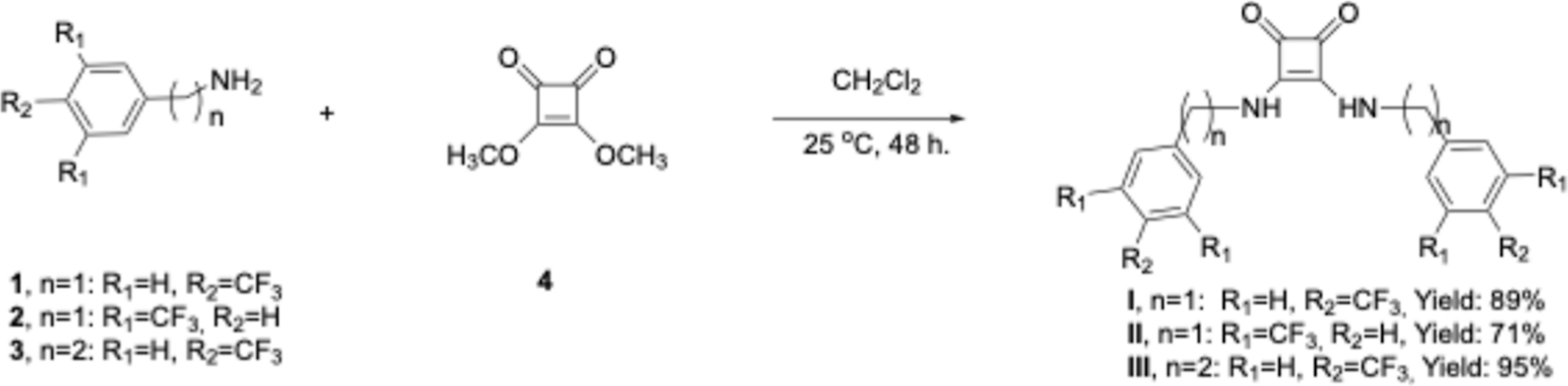
Synthesis of Squaramide Receptors **I**–**III**

### ^1^H NMR Titrations of Squaramides **I**–**III** with TBA-Cl

^1^H NMR titrations of squaramides **I**–**III** with the addition of TBA-Cl from 0.2 to 20.0 equiv were performed, and the variations in the NH proton signals of the squaramides were monitored after addition of each equivalent. The spectra and chemical shifts in NH proton peaks for the titration of squaramides **I**–**III** with TBA-Cl are demonstrated in Figure 2. As evident from the spectra, the NH signals shift downfield upon addition of TBA-Cl, indicating the formation of hydrogen bonds between the Cl^–^ anion and NH groups. The difference in the chemical shift for squaramide **I**, in which one –CF_3_ group on the aromatic ring and one –CH_2_– unit between the aromatic ring and the –NH, is 1.58 ppm. This difference decreased to 1.52 ppm for the squaramide **II** with two –CF_3_ groups on the aromatic ring and one –CH_2_– unit between the aromatic ring and the –NH. The chemical shift difference is 1.53 ppm in squaramide **III**, in which one –CF_3_ group is on the aromatic ring and two –CH_2_– units between the aromatic ring and the –NH. As a result of the ^1^H NMR spectroscopic titrations, variations in the NH proton chemical shifts of the squaramides followed the order SQ **I** > SQ **III** > SQ **II**.

**Figure 2:**
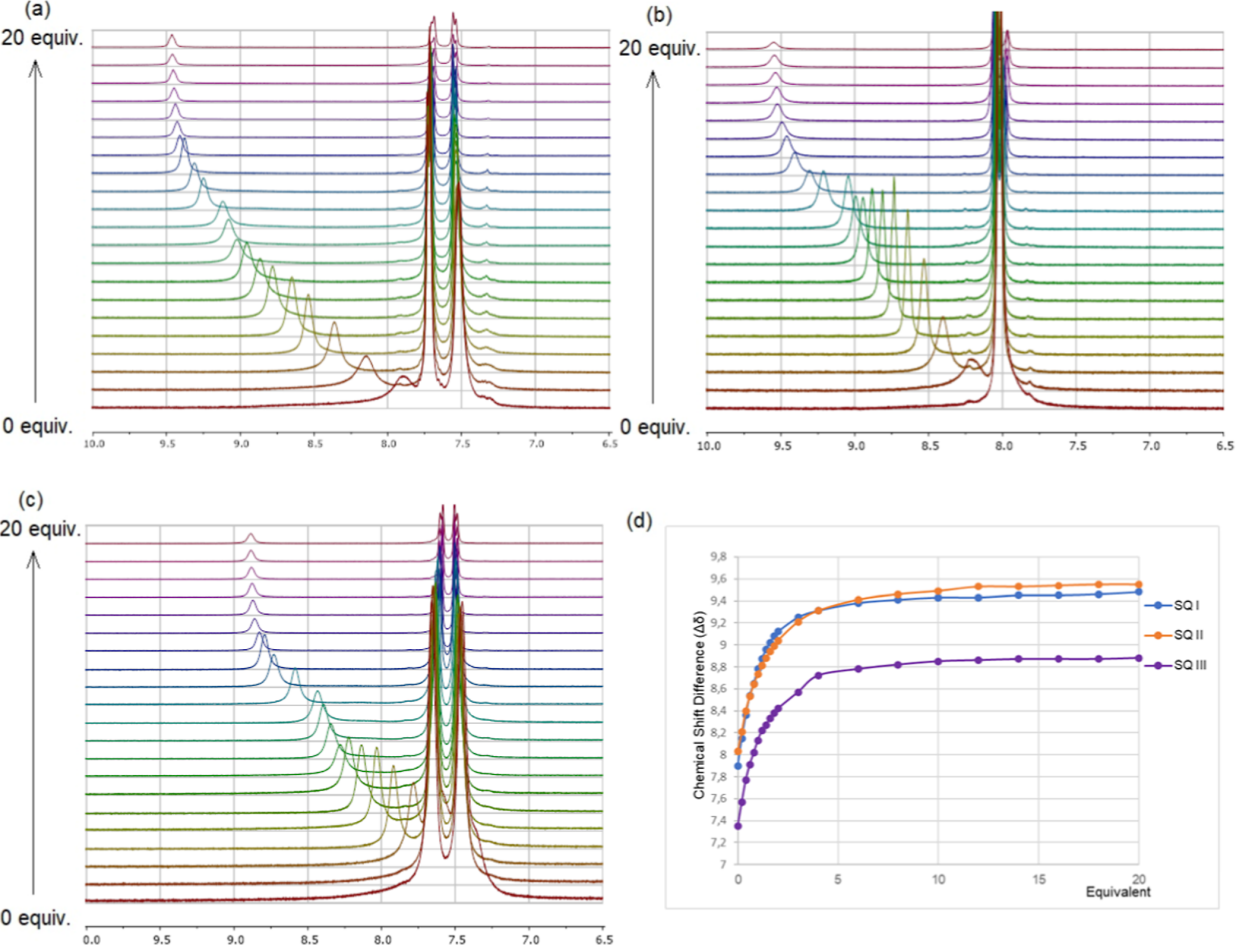
^1^H NMR titration spectra of squaramide **I** (a), squaramide **II** (b), and squaramide **III** (c) upon addition of TBA-Cl (0–20 equiv) in [d_6_] DMSO at 25 °C; (d) chemical shift changes of the NH protons as a function of the equivalents of TBA-Cl added.

In addition to TBA-Cl, ^1^H NMR spectroscopic titration was carried out with squaramide **I** and TBA-Br, TBA-I, and TBA-ClO_4_ salts (guests), independently. The downfield chemical shift of NH protons for squaramide **I** when titrated with TBA-Br was found as 0.63 ppm (Figure 3).

**Figure 3:**
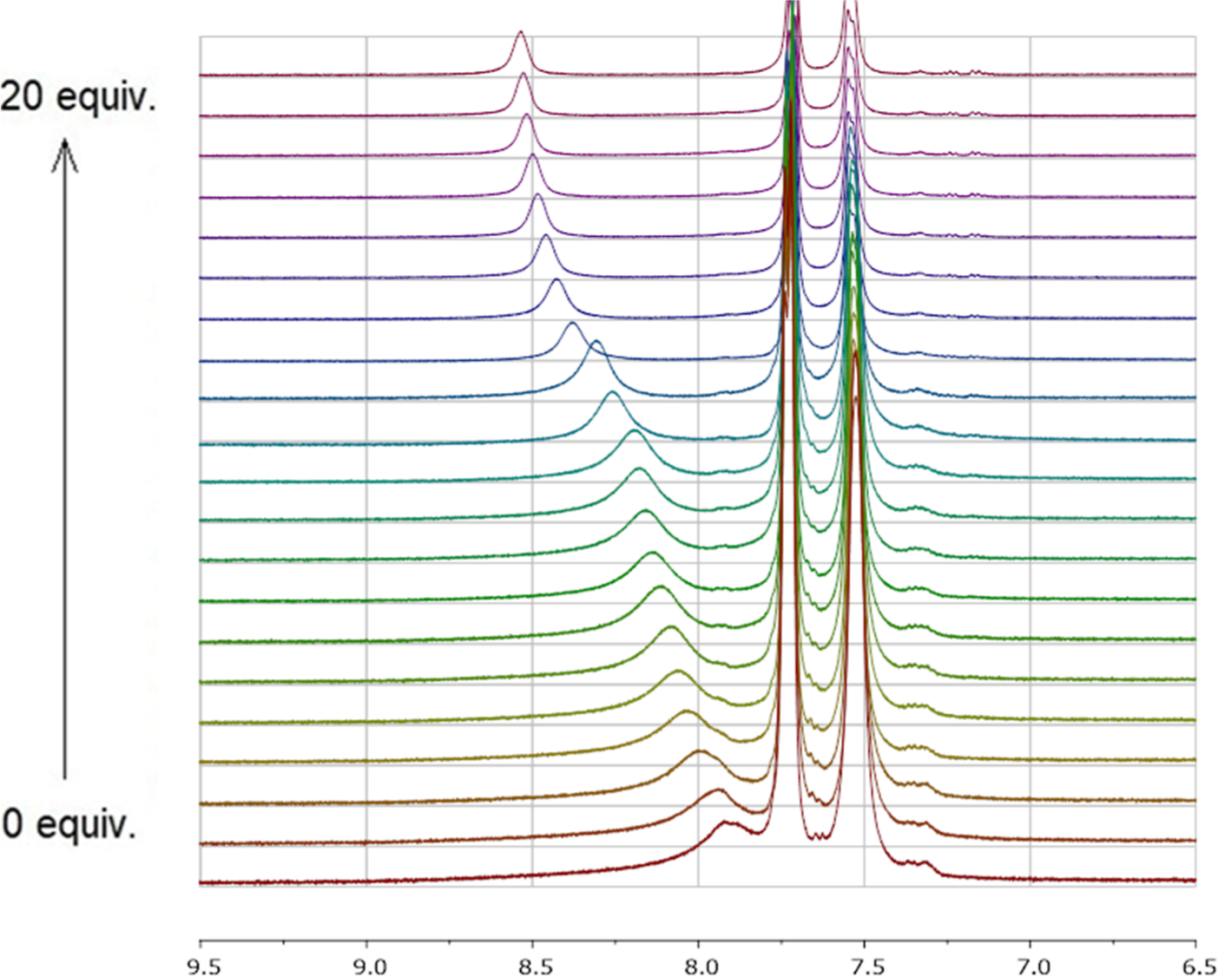
^1^H NMR titration spectrum of squaramide **I** upon addition of TBA-Br (0–20 equiv) in [d_6_] DMSO at 25 °C.

Titrations of squaramide **I** with TBA-I or TBA-ClO_4_ did not produce any observable changes in the NH proton chemical shifts. This behavior can be attributed to differences in the anion size as the hydrogen-bonding affinity decreases with increasing atomic diameter.

### Binding Properties of Squaramides

The DynaFit^39–41^ and BindFit^42–44^ programs based on a 1:1 binding model were used to analyze the NH protons chemical shifts obtained from the ^1^H NMR titrations of squaramides **I**–**III** with TBA-Cl.^45^ Fitting the data with the 1:1 model in DynaFit generated fit plots of the NH proton shifts versus TBA-Cl concentration (Figure 4 a–c), residuals (Figure 4 d–f), and relative sums of squares (SSQ/SSQ min.) (Figure 4 g–i).

**Figure 4:**
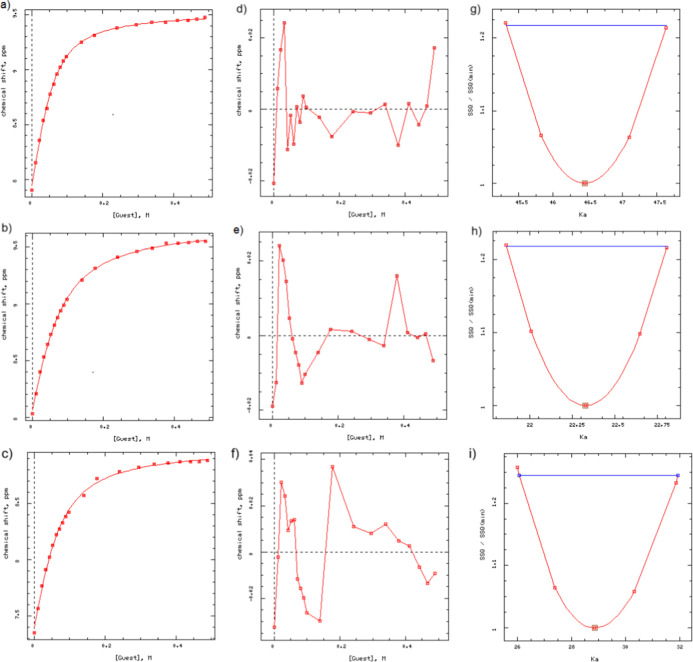
Plot of downfield shifts of NH protons versus TBACl concentration during ^1^H NMR titration in DMSO-*d*_6_ at (a) δ = 7.90 ppm in squaramide **I**, (b) δ = 8.03 ppm in squaramide **II**, (c) δ = 7.35 ppm in squaramide **III**; residual (d) δ = 7.90 ppm in squaramide **I**, (e) δ = 8.03 ppm in squaramide **II**, and (f) δ = 7.35 ppm in squaramide **III**; relative sum of squares (SSQ/SSQ min). (g) *K*_a_ = 47.65 ± 1.30 M^–1^ (NH-proton at δ = 7.90 ppm), (h) *K*_a_ = 22.82 ± 0.65 M^–1^ (NH-proton at δ = 8.03 ppm), and (i) *K*_a_ = 28.90 ± 1.40 M^–1^ (NH-proton at δ = 7.35 ppm).

Based on a 1:1 binding model, *K*_a_ values for Cl^–^ anion binding were found as 47.65 ± 1.30 M^–1^ for squaramide **I**, 22.82 ± 0.65 M^–1^ for squaramide **II**, and 28.90 ± 1.40 M^–1^ for squaramide **III** via DynaFit analysis^39^ (Figure 4). Accordingly, the binding strength of the squaramides toward the Cl^–^ anion, as reflected by their *K*_a_ values, was found to follow the trend SQ **I** > SQ **III** > SQ **II**.

In addition, *K*_a_ values were determined using BindFit software, a widely utilized tool in supramolecular anion recognition studies. The stoichiometric *K*_a_ values for Cl^–^ anion binding were found as 49.78 ± 3.36 M^–1^ for squaramide **I**, 23.90 ± 2.70 M^–1^ for squaramide **II**, and 28.80 ± 5.04 M^–1^ for squaramide **III** via BindFit analysis (Figures 5–7) (see Figure 6).

**Figure 5:**
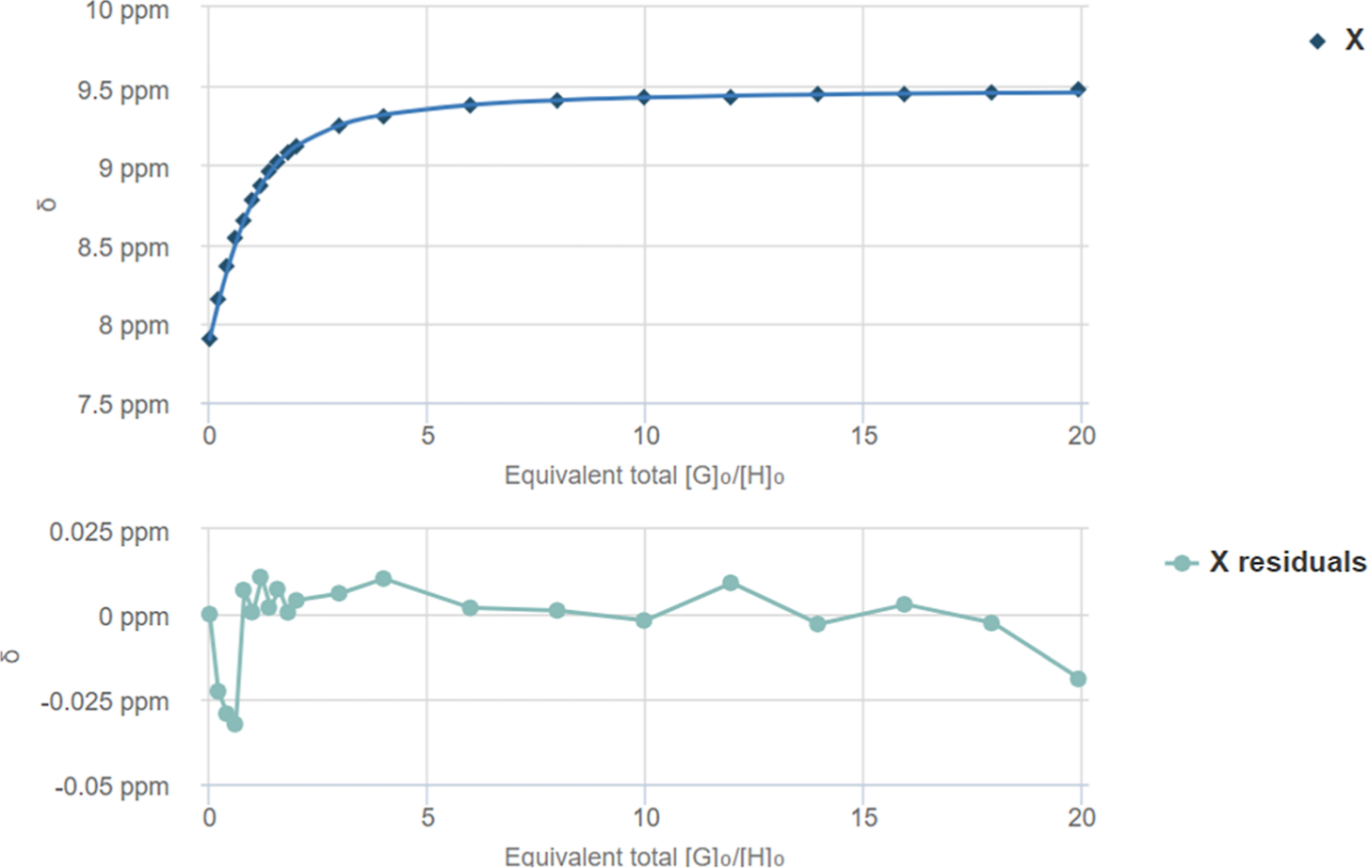
Fit plot for the NH proton at δ = 7.90 ppm. *K*_a_ = 49.78 ± 3.36 M^–1^ and residual according to BindFit 1:1 analysis of ^1^H NMR titration between squaramide **I** and TBA-Cl.

**Figure 6:**
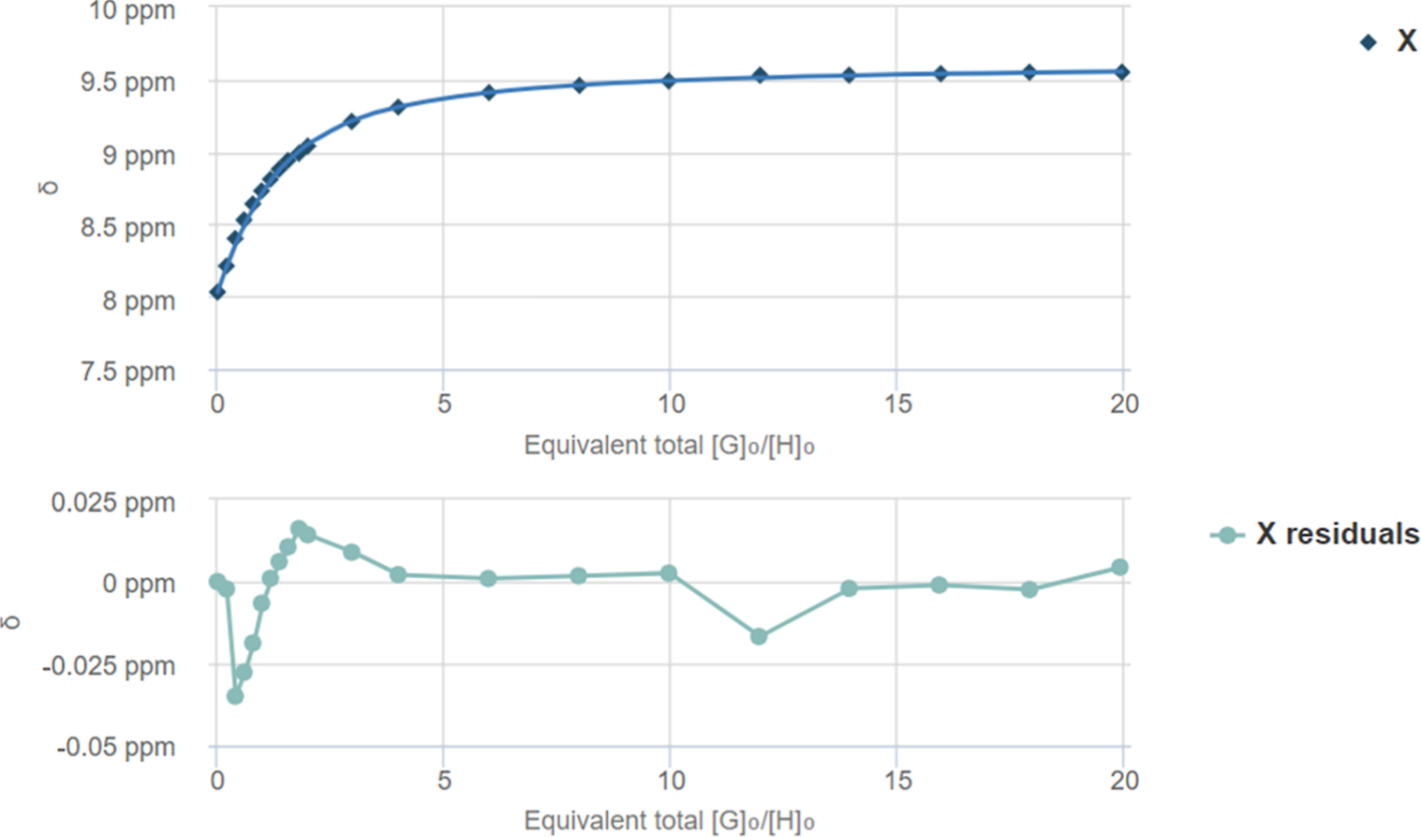
Fit plot for the NH proton at δ = 8.03 ppm. *K*_a_ = 23.90 ± 2.70 M^–1^ and residual according to BindFit 1:1 analysis of ^1^H NMR titration between squaramide **II** and TBA-Cl.

**Figure 7:**
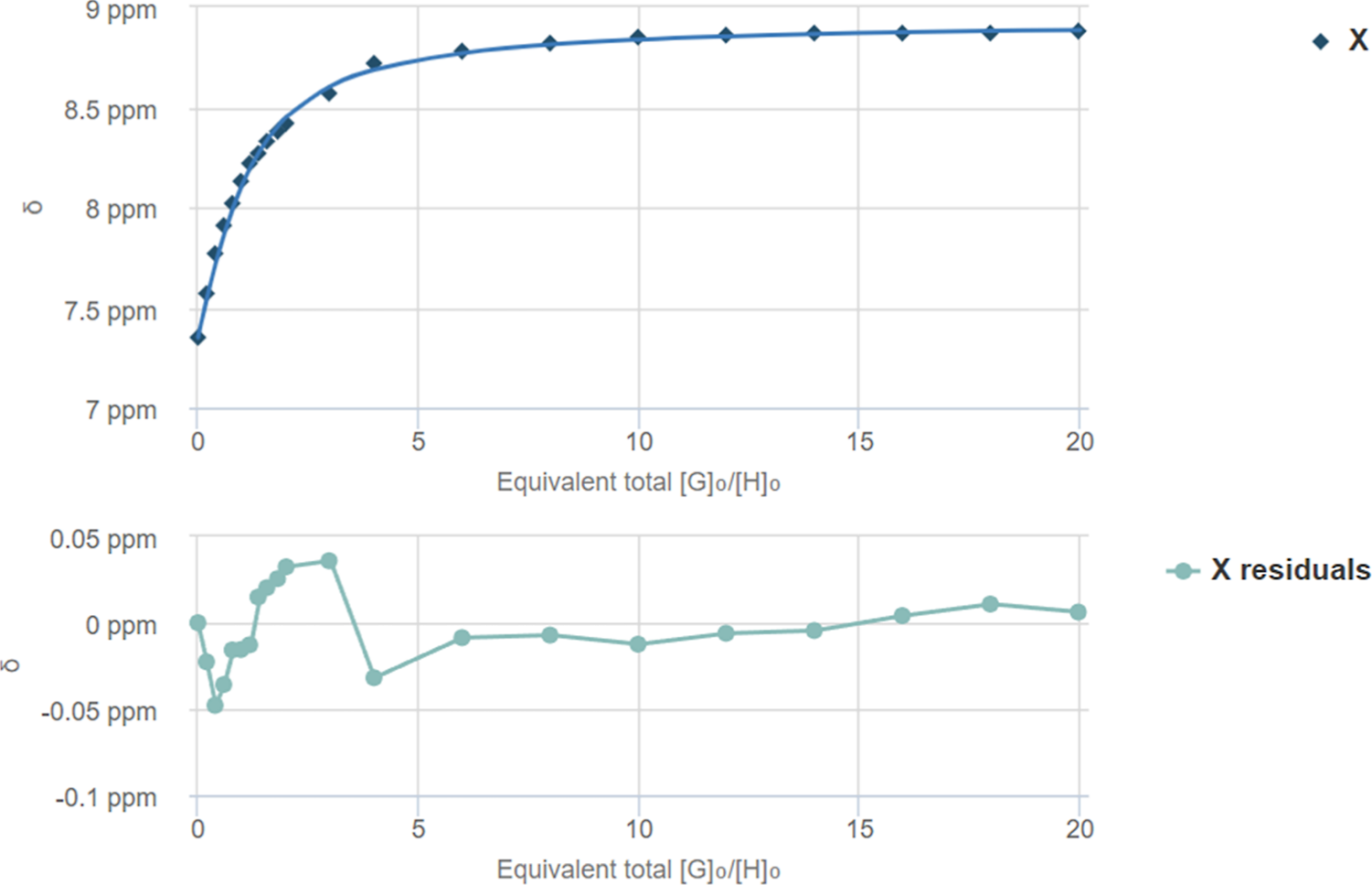
Fit plot for the NH proton at δ = 7.35 ppm. *K*_a_ = 28.80 ± 5.04 M^–1^ and residual according to BindFit 1:1 analysis of ^1^H NMR titration between squaramide **III** and TBA-Cl.

Table 1 summarizes the binding constants (*K*_a_ ± SD) of SQs **I−III** for the Cl^–^ anion, as determined using DynaFit^39–41^ and BindFit^42–44^ software according to a 1:1 binding model. As shown in Table 1, moderate *K*_a_ values for SQs **I**-**III** and Cl^–^ anion binding were obtained. Solvent selection can significantly influence the receptor–anion interaction strength. Because DMSO-*d*_6_ is capable of forming hydrogen bonds with squaramides, it may have acted as a competing species, thereby weakening the interactions between squaramides and the anions.^34,35^ Nevertheless, DMSO-*d*_6_ was used in this study because of the limited solubility of the squaramides.

**Table 1:** Binding Constants (*K*_a_ Values) of SQs **I**-**III** Calculated According to DynaFit1:1 and BindFit1:1 Software

		*K*_a_ (M^–1^) ± SD		
anion	model	Sq. **I**	Sq. **II**	Sq. **III**
Cl^–^	DynaFit 1:1	47.65 ± 1.30	22.82 ± 0.65	28.90 ± 1.40
Cl^–^	BindFit 1:1	49.78 ± 3.36	23.90 ± 2.70	28.80 ± 5.04

When the aromatic group is directly attached to the NH moiety without a methylene spacer, Cl^–^ anion binding constants (*K*_a_) were reported to be 458 M^–1^ for the squaramide derivative bearing a single CF_3_ substituent on the aromatic ring and 643 M^–1^ for the derivative bearing two CF_3_ substituents.^15^ In comparison with these literature values, the results of this study indicate that the introduction of a methylene spacer led to a decrease in the *K*_a_ value, contrary to our initial expectations. Furthermore, the presence of two CF_3_ substituents on the aromatic ring was shown to enhance Cl^–^ anion binding affinity in the literature,^15^ a decrease in the *K*_a_ value was observed in our system.

According to DynaFit analysis, the stoichiometric *K*_a_ value for the interaction between squaramide **I** and Br^–^ anion was determined to be 10.24 ± 3.20 M^–1^ (Supporting Information, Figure S11), whereas BindFit analysis resulted in a value of 6.14 ± 1.94 M^–1^ (Supporting Information, Figure S12). These results demonstrate that the binding strength of squaramide **I** toward the Cl^–^ anion is greater than that toward the Br^–^ anion.

### Job Plot

The maximum host fractions (*X*_max_) for SQs **I**–**III**, obtained from Job plot analyses with TBA-Cl, were determined to be 0.50, 0.46, and 0.45, respectively (Figure 8a–c). Based on the equation *Y*= (1/*X*_max_)-1,^44^ the host-to-guest (H/G) stoichiometric ratios were calculated to be 1:1 (XY) for SQ **I** and the Cl^–^ anion, a combined 1:1 (XY) and 1:2 (XY_2_) for SQ **II** or SQ **III** and Cl^–^ anion. Similarly, for SQ **I** titrated with TBA-Br, a combined 1:1 and 1:2 binding model was indicated, with an *X*_max_ value of 0.39 (Figure 8d). Accordingly, both DynaFit and BindFit analyses using a 1:1 binding model were employed to calculate the *K*_a_ values, while Job plot analysis was used to determine the binding stoichiometry.

**Figure 8:**
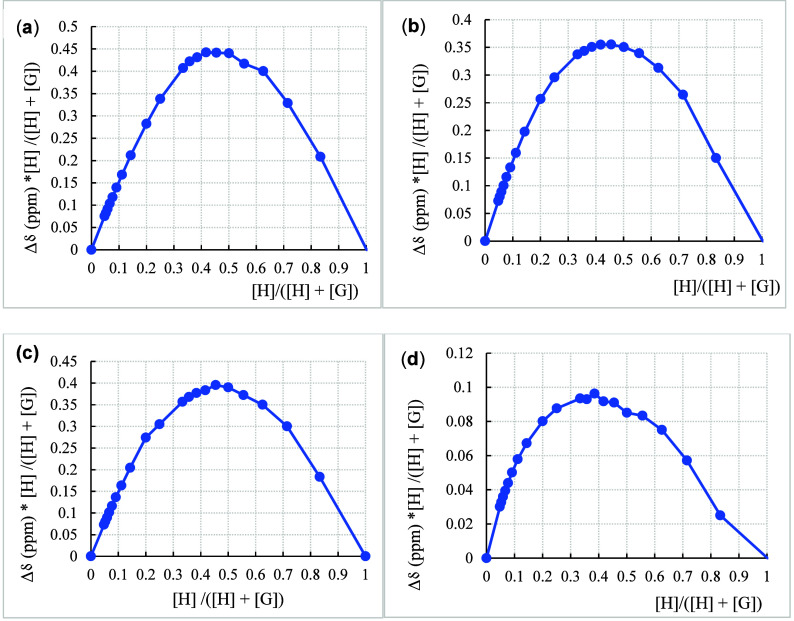
Job plots of ^1^H NMR spectroscopic TBA-Cl titration with squaramide **I** (*X*_max_: 0.50) (a); squaramide **II** (*X*_max_: 0.46) (b); squaramide **III** (*X*_max_: 0.45) (c); TBA-Br titration with squaramide **I** (*X*_max_: 0.39) (d).

## Conclusion

In this study, three different squaramide derivatives (SQ **I**–**III**) were synthesized and their anion-binding capacities were evaluated via titrations with TBA-Cl. A comparison of the binding constants toward the Cl^–^ anion revealed the following order of affinity: SQ **I** > SQ **III** > SQ **II**. The results indicated that both the number of CF_3_ substituents on the aromatic ring and the length of the methylene spacer (CH_2_) between the aromatic ring and the amide group significantly influence the anion binding strength. The highest binding affinity was observed for SQ **I**, which contains a single CF_3_ group on the aromatic ring and one CH_2_ group between the aromatic ring and the amide moiety. Following the identification of SQ **I** as the most effective Cl^–^ anion binder, additional titrations using SQ **I** were carried out using TBA-Br, TBA-I, and TBA-perchlorate to assess its interactions with other anions. While SQ **I** exhibited weak binding with Br^–^, no significant interaction was observed with either I^–^ or perchlorate anions.

## Supplementary Material



## Notes

^1^H NMR,^13^C NMR, and ATR-FTIR spectra of all squaramides, LC/MS-TOF spectrum of a novel squaramide, stack plots of ^1^H NMR spectra of addition of TBA-Br to SQ **I**, example for 1:1 binding model DynaFIT Script, graphs of DynaFit 1:1 analysis, and graphs of BindFit 1:1 analysis (PDF)
