# Supplementary material for: Investigation of Chloride Binding Abilities of Symmetric Squaramides
Source: ACS Omega. 2026 Mar 23;11(13):20936–44. doi: 10.1021/acsomega.5c13503 (PMC13063069; doi:10.1021/acsomega.5c13503)
Supplement: Supplementary file 1 [file ao5c13503_si_001.pdf]

Investigation of Chloride Binding Abilities of Symmetric Squaramides

Serap Mert[1-3], Sude Minel Özger[4], Ayşenur Vardar[1], and Özden Erdebil[2]*

[1] Department of Chemistry, Faculty of Arts and Sciences, Kocaeli University, 41001, Kocaeli, Türkiye

[2] Department of Polymer Sci. and Technol., Institute of Natural and Applied Sciences, Kocaeli University, 41001, Kocaeli, Türkiye

[3] Center for Stem Cell and Gene Therapies Res. and Pract., Kocaeli University, 41001, Kocaeli, Türkiye

[4] Department of Chemical Engineering, Engineering Faculty, Kocaeli University, 41001, Kocaeli, Türkiye

*Email: serap.mert@kocaeli.edu.tr

^{1}H NMR, ^{13}C NMR, ATR-FTIR, and LC/MS-TOF spectra of squaramides

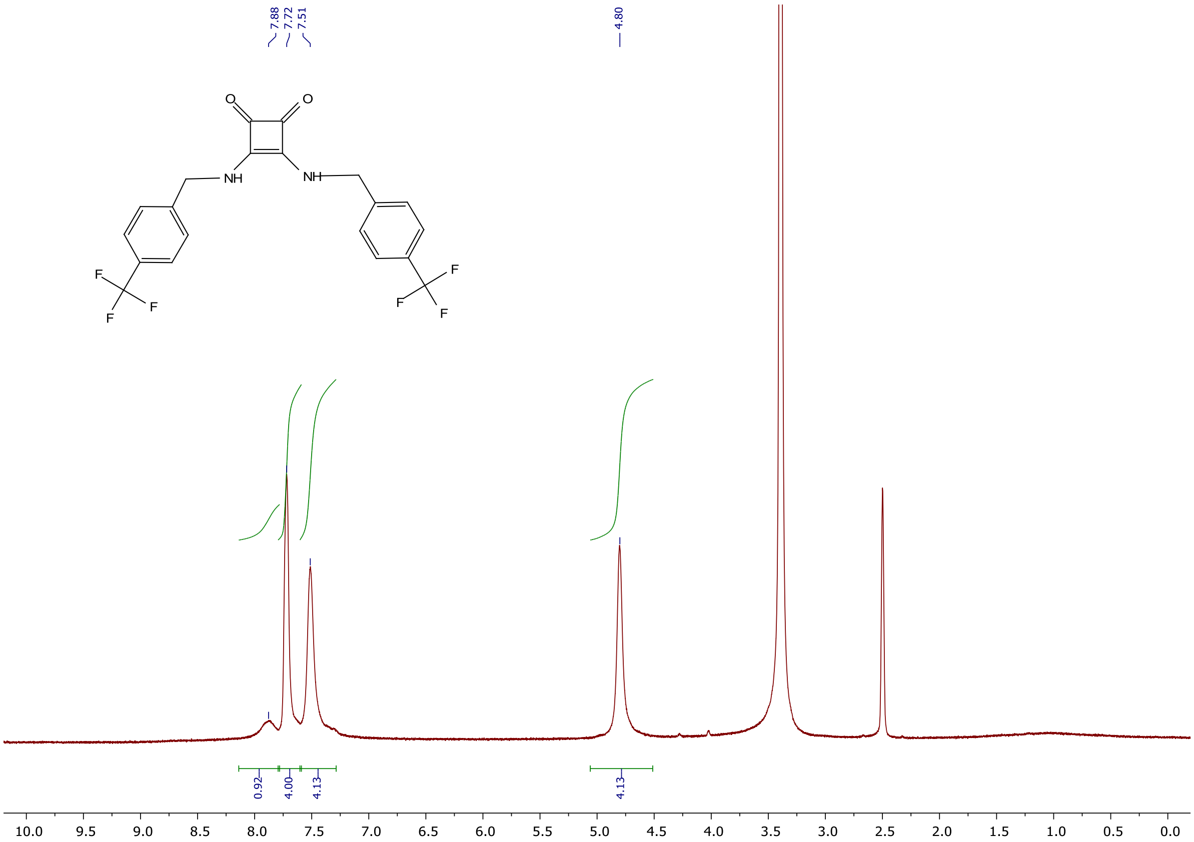

Figure S1. ^{1}H-NMR Spectrum of squaramide **I**

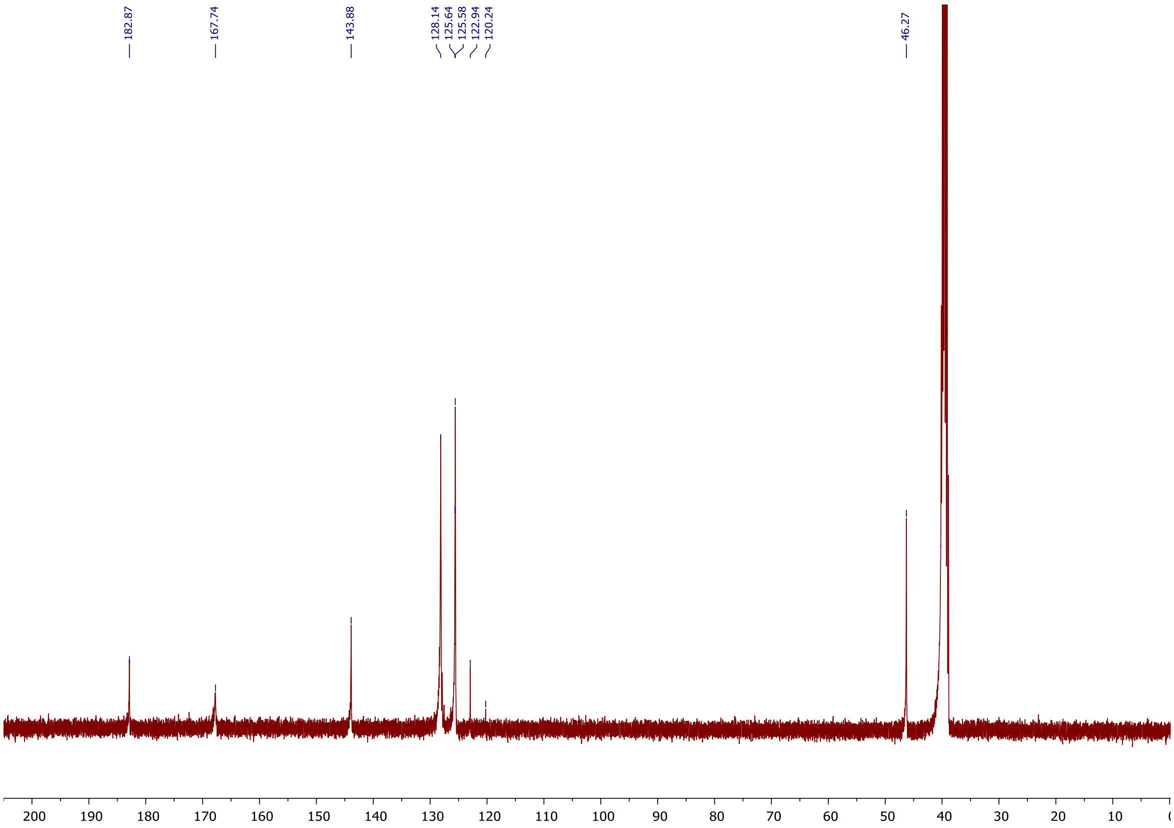

Figure S2. ^{13}C-NMR Spectrum of squaramide **I**

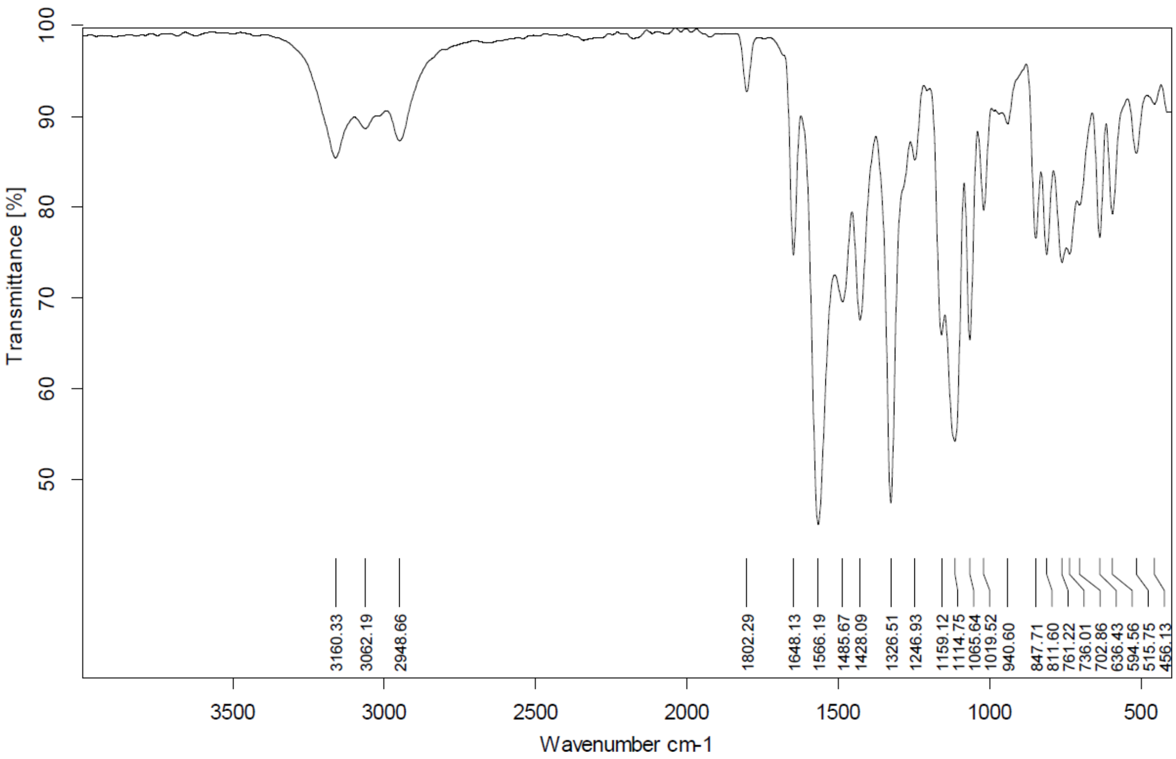

Figure S3. ATR-FTIR spectrum of squaramide **I**

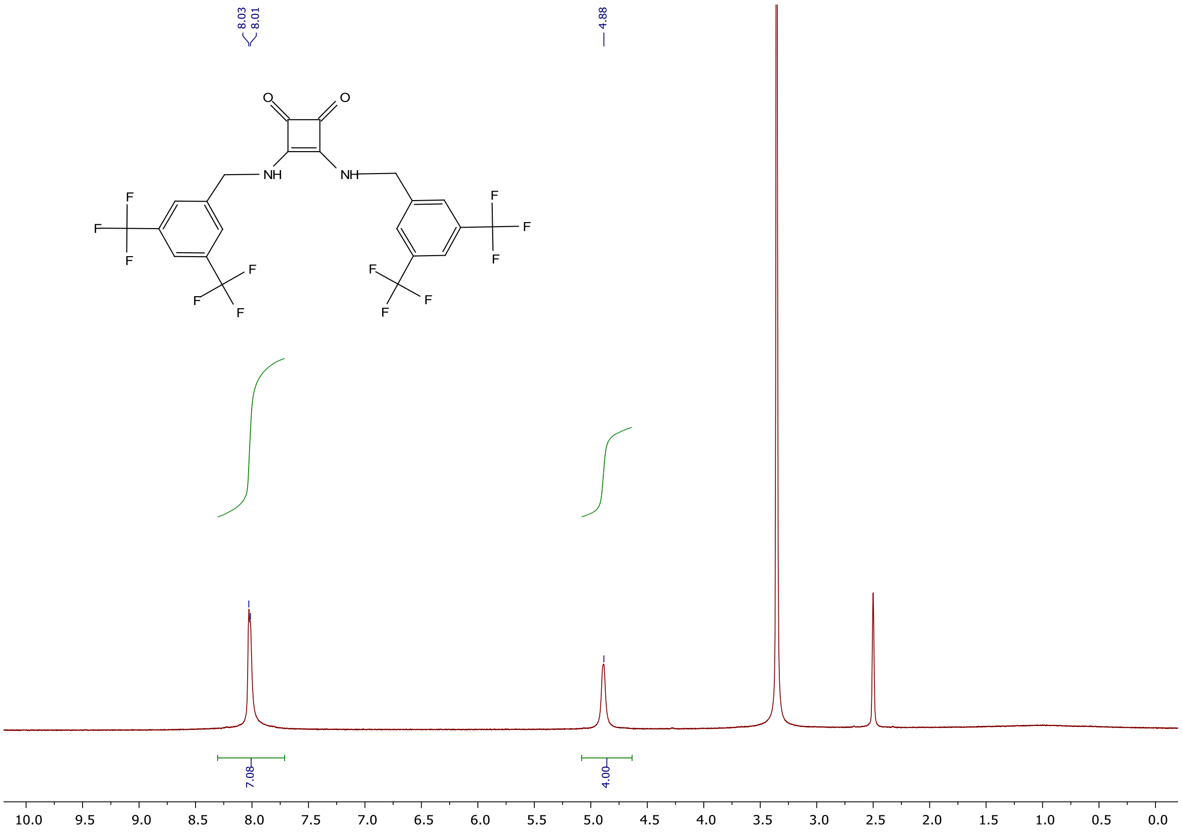

Figure S4. ^{1}H-NMR Spectrum of squaramide **II**

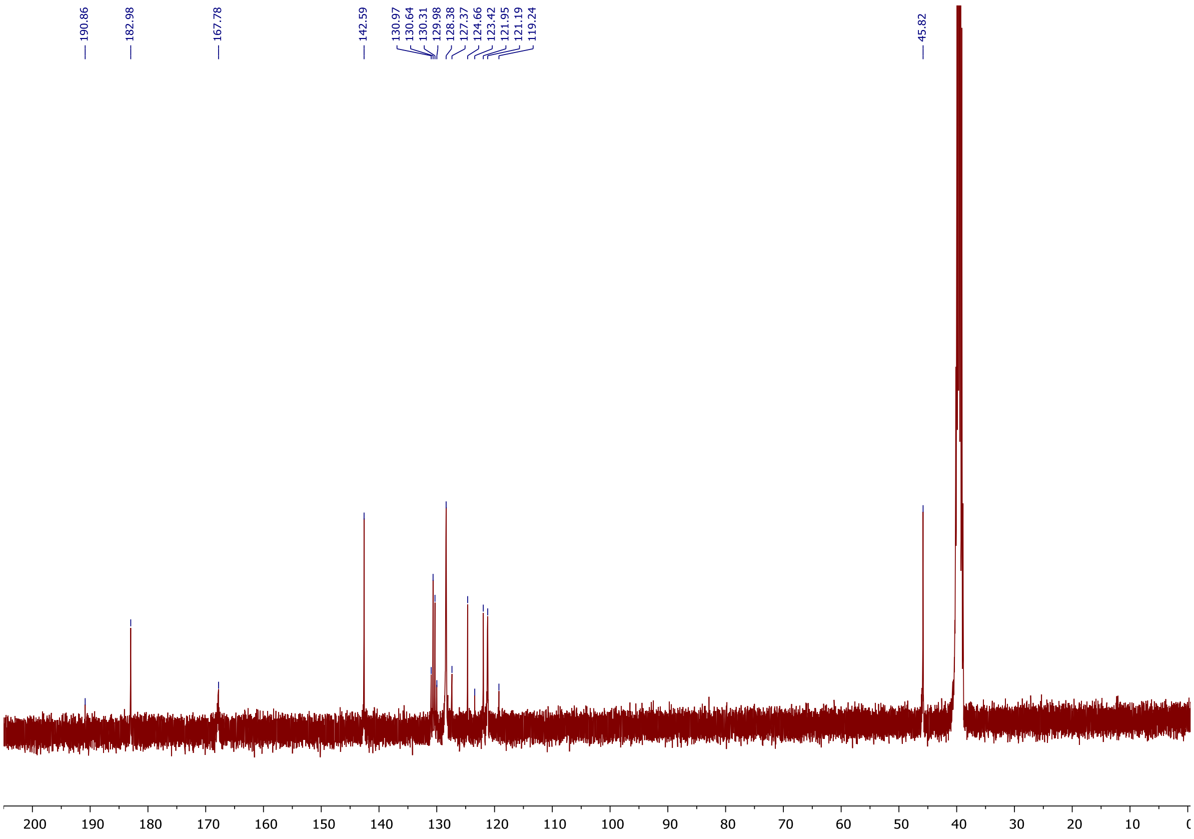

Figure S5. ^{13}C-NMR Spectrum of squaramide **II**

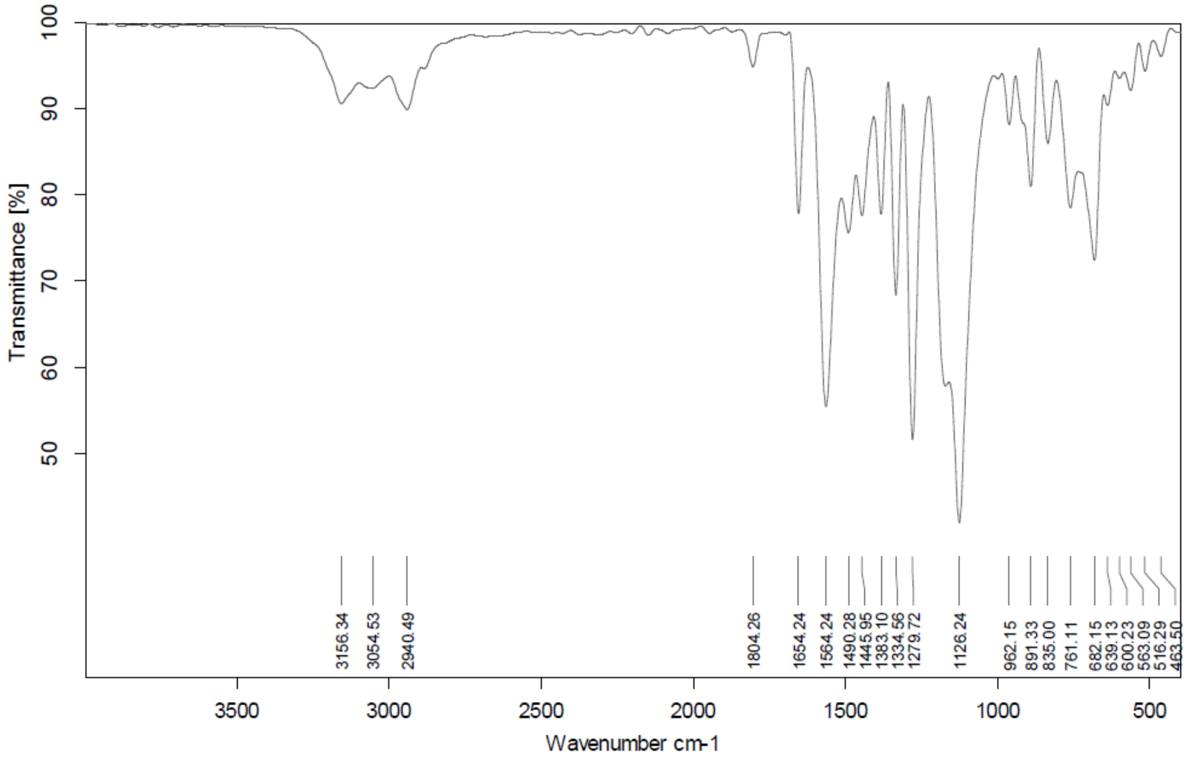

Figure S6. ATR-FTIR spectrum of squaramide **II**

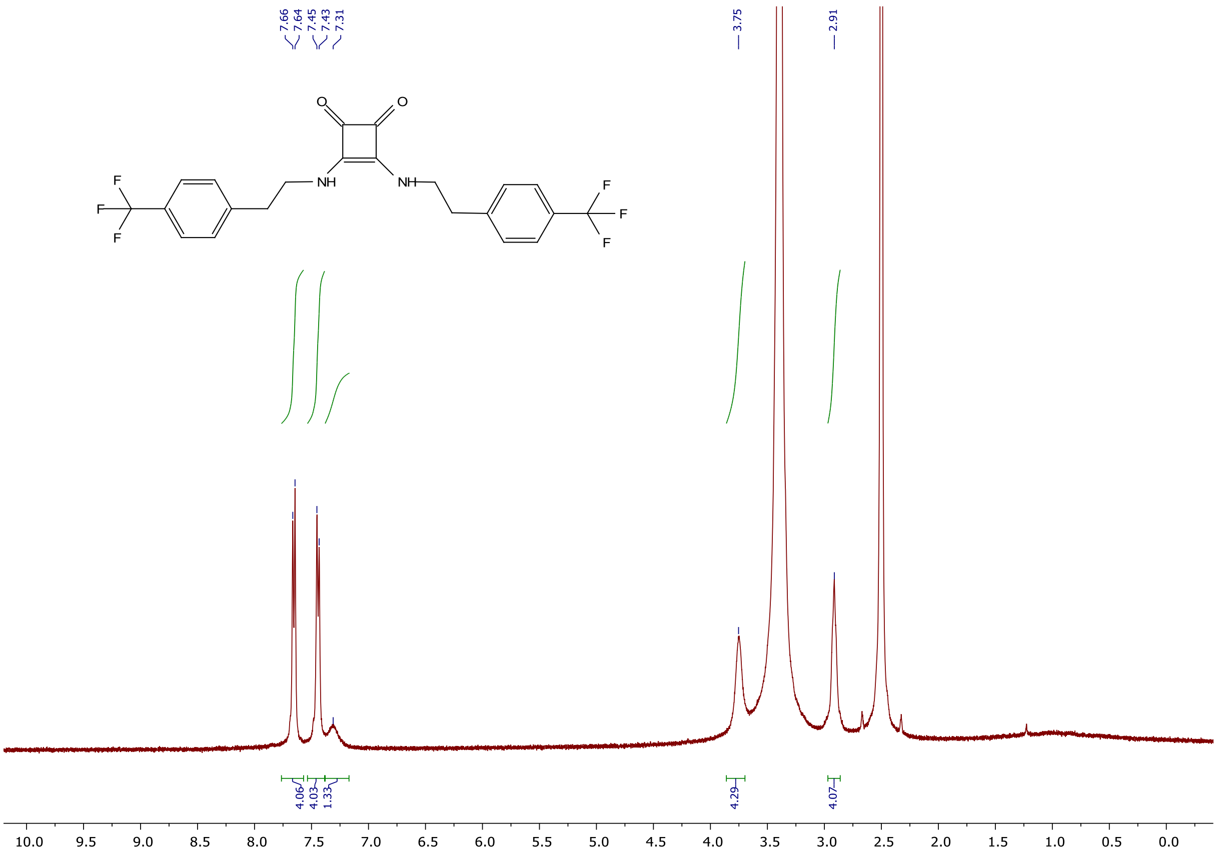

Figure S7. ^{1}H-NMR Spectrum of Squaramide **III**

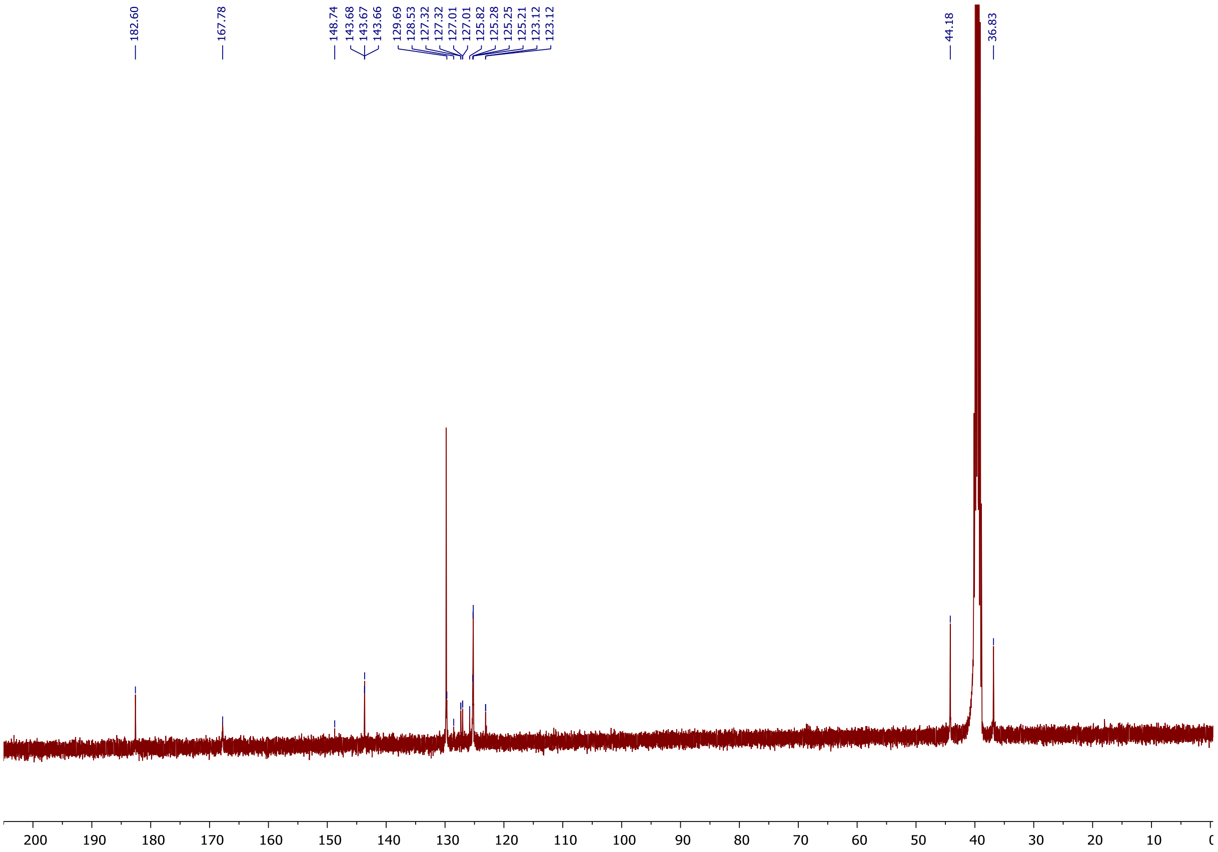

Figure S8. ^{13}C-NMR Spectrum of Squaramide **III**

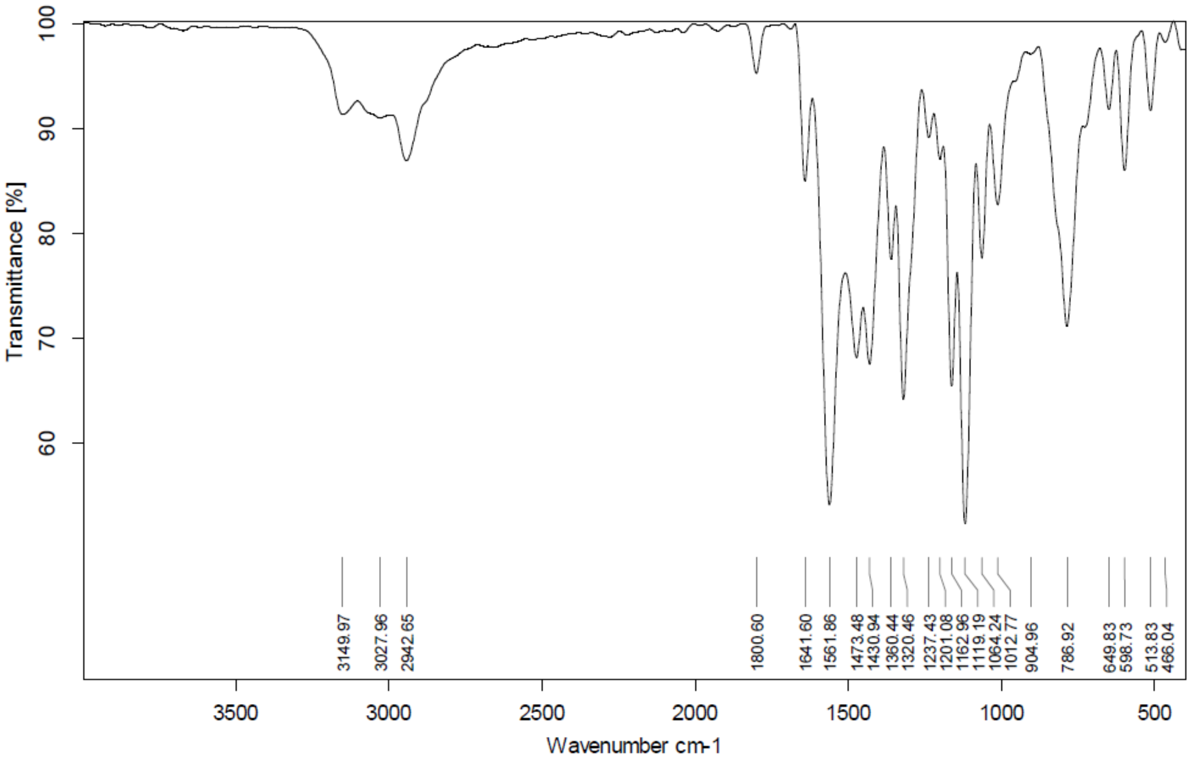

Figure S9. ATR-FTIR spectrum of Squaramide **III**

Qualitative Analysis Report

**Data Filename**	Sample23.d	**Sample Name**	Sample23
**Sample Type**	Sample	**Position**	P1-C5
**Instrument Name**	Instrument 1	**User Name**	Oguzhan DALKILIC
**Acq Method**	ESI pos.m	**Acquired Time**	12/14/2022 2:55:43 PM
**IRM Calibration Status**	Success	**DA Method**	Default.m
**Comment**			

**Sample Group**		**Info.**	
**Stream Name**	LC 1	**Acquisition SW Version**	6200 series TOF/6500 series Q-TOF B.08.00 (B8058.0)

User Chromatograms

Fragmentor Voltage 90 **Collision Energy** 0 **Ionization Mode** ESI

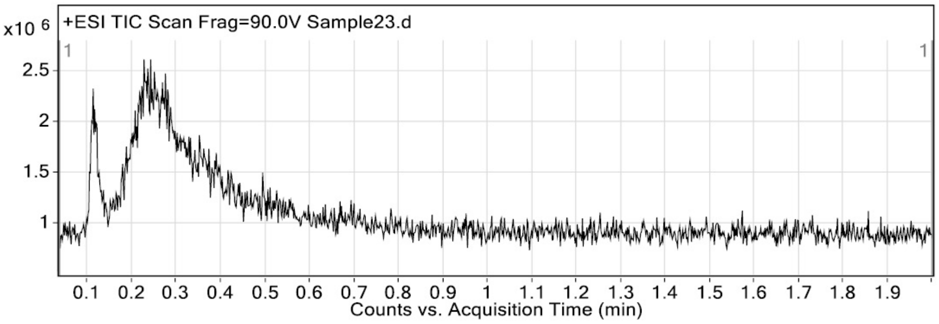

User Spectra

Fragmentor Voltage	Collision Energy	Ionization Mode
90	0	ESI

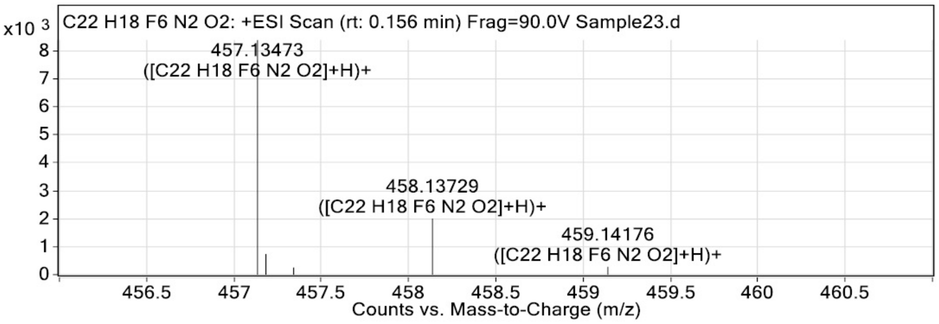

Peak List

m/z	z	Abund	Formula	Ion
457.13473	1	8378.85	C22 H18 F6 N2 O2	(M+H)+
457.18391		735.07		
458.13729	1	2000.58	C22 H18 F6 N2 O2	(M+H)+

Formula Calculator Element Limit

Element	Min	Max
C	3	22
H	0	18
O	0	2
N	0	2
F	0	6

Formula Calculator Results

Formula	Best	Mass	Tgt Mass	Diff (ppm)	Ion Species	Score
C22 H18 F6 N2 O2	DOĞRU	456,12736	456,12725	-0,25	C22 H19 F6 N2 O2	99,37

--- End Of Report ---

Figure S10. LC/MS-TOF Spectrum of Squaramide **III**

Example 1:1 Binding Model DYNAFIT Script[1]

```
[task]
   data = equilibria
   task = fit

[mechanism]
   H + G <==> H.G      :      Ka    assoc

[constants]
   Ka =  ??

[responses]
   intensive ; ... NMR chemical shifts

[data]
   variable G, H
   plot titration ; ... [H] and [G] are varied simultaneously
   graph 1H ; proton chemical shifts
   set aH | response H = 7.90 ?, H.G = 9.48 ?

[output]
   directory ./examples/pt03/NMR/output/aa
[settings]
{Output
   XAxisLabel = [Guest], M
   YAxisLabel = chemical shift, ppm
[end]
```

Graphs of DynaFit 1:1 analysis

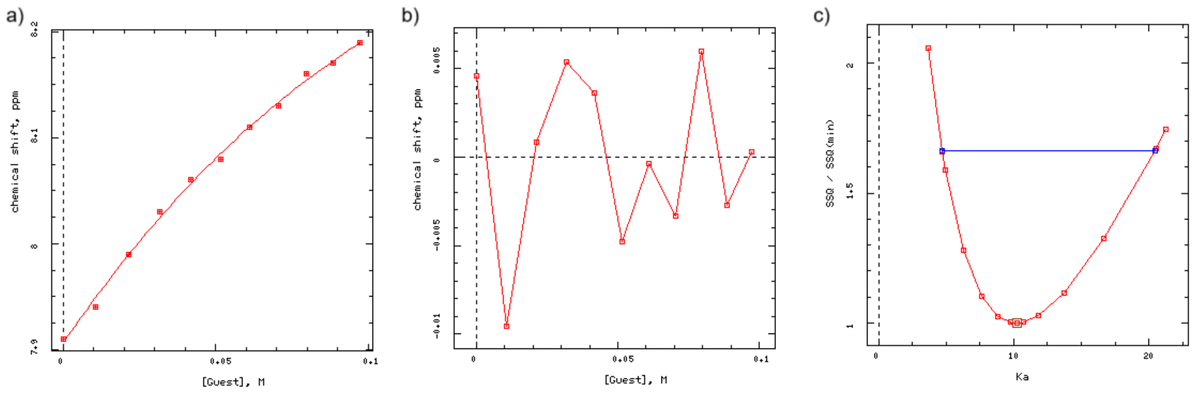

Figure S11. Plot of downfield shifts of NH protons (7.90 ppm) of squaramide **I** versus TBA-Br concentration during ^{1}H NMR titration in d_6-DMSO **(a)**, Residual **(b),** Relative sum of squares (SSQ/SSQ min.), K_a = 10.24 ± 3.20 M^{-1} (NH-proton at δ = 7.90 ppm) **(c)**

Graphs of BindFit 1:1 analysis

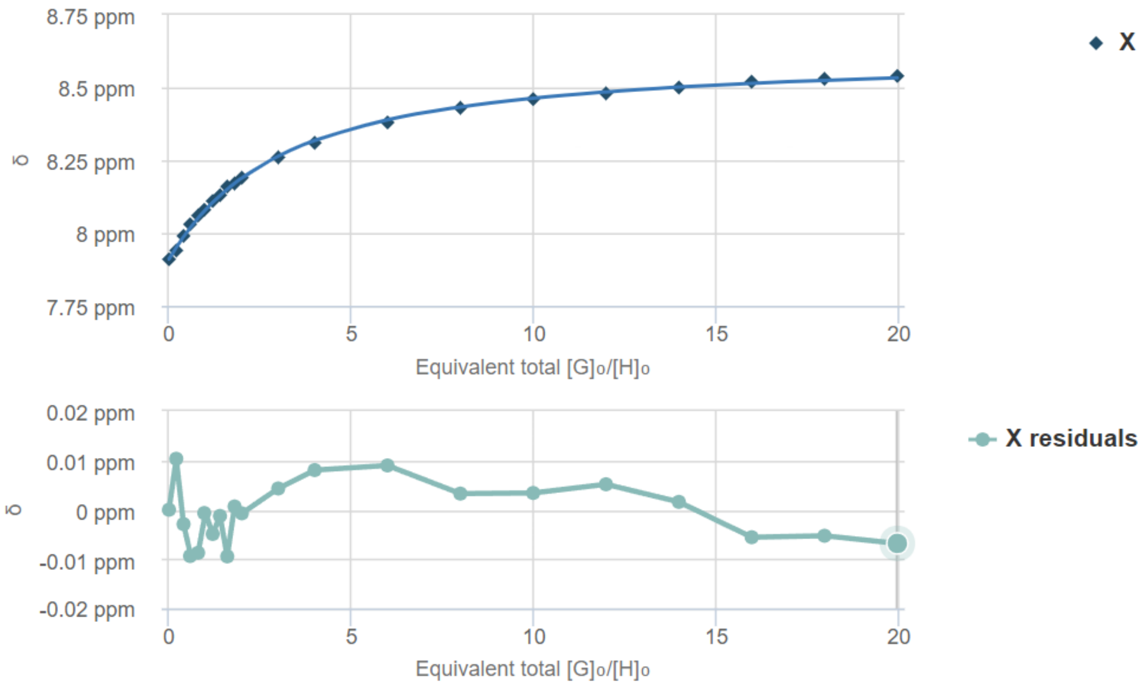

Figure S12. Fitplot for NH proton at δ = 7.90 ppm. K_a = 6.14 ± 1.94 M^{-1} and Residual according to BindFit 1:1 analysis of ^{1}H NMR titration between squaramide **I** and TBA-Br

REFERENCES

1. Kuzmic, P., DynaFit-a software package for enzymology. *Methods Enzymol.* **2009,** *467*, 247-280.
